# Optimal thresholds and key parameters for predicting influenza A virus transmission events in ferrets

**DOI:** 10.1038/s44298-024-00074-w

**Published:** 2024-12-09

**Authors:** Troy J. Kieran, Xiangjie Sun, Taronna R. Maines, Jessica A. Belser

**Affiliations:** Influenza Division, Centers for Disease Control and Prevention, Atlanta, GA USA

**Keywords:** Influenza virus, Viral transmission

## Abstract

Although assessments of influenza A virus transmissibility in the ferret model play a critical role in pandemic risk evaluations, few studies have investigated which virological data collected from virus-inoculated animals are most predictive of subsequent virus transmission to naïve contacts. We compiled viral titer data from >475 ferrets inoculated with 97 contemporary IAV (including high- and low-pathogenicity avian, swine-origin, and human viruses of multiple HA subtypes) that served as donors for assessments of virus transmission in the presence of direct contact (DCT) or via respiratory droplets (RDT). A diversity of molecular determinants, clinical parameters, and infectious titer measurements and derived quantities were examined to identify which metrics were most statistically supported with transmission outcome. Higher viral loads in nasal wash (NW) specimens were strongly associated with higher transmission frequencies in DCT, but not RDT models. However, viruses that reached peak titers in NW specimens early (day 1 p.i.) were strongly associated with higher transmission in both models. Interestingly, viruses with ‘intermediate’ transmission outcomes (33–66%) had NW titers and derived quantities more similar to non-transmissible viruses (<33%) in a DCT setting, but with efficiently transmissible viruses (>67%) in a RDT setting. Machine learning was employed to further assess the predictive role of summary measures and varied interpretation of intermediate transmission outcomes in both DCT and RDT models, with models employing these different thresholds yielding high performance metrics against both internal and external datasets. Collectively, these findings suggest that higher viral load in inoculated animals can be predictive of DCT outcomes, whereas the timing of when peak titers are detected in inoculated animals can inform RDT outcomes. Identification that intermediate transmission outcomes should be contextualized relative to the transmission mode assessed provides needed refinement towards improving interpretation of ferret transmission studies in the context of pandemic risk assessment.

## Introduction

Among the three necessary criteria for a virus to cause a pandemic in humans (little to no preexisting immunity in the human population, ability to cause illness in humans, and capacity for sustained human-to-human transmissibility)^1^, it is the latter typically lacking among zoonotic-origin influenza A viruses (IAV) with pandemic potential. Intensive study to elucidate molecular determinants of IAV transmissibility by the airborne route have identified key residues in the HA and PB2 proteins (including receptor binding preferences and polymerase activity) known to modulate this multifactorial trait^2^. However, while assessments of other modes of transmission (such as direct or indirect contact with contaminated surfaces or fomites) are known to contribute to onward human infection^3–5^, few studies have assessed molecular determinants associated with non-airborne transmission routes, or investigated relative differences between transmission modes in a head-to-head fashion. As the ferret model supports replication of both human and zoonotic-origin IAV, this species represents the most frequently employed small mammalian species to study IAV transmissibility in the context of pandemic risk assessment^6^. Well-established models of virus transmission between ferrets via respiratory droplets (RDT) and in the presence of direct contact (DCT) are employed across laboratories worldwide^7^, as these setups permit a high degree of flexibility and manipulation to achieve specific experimental aims, with results similar to transmission rates observed in humans^8^.

Machine learning (ML) algorithms make predictions from data by identifying patterns and connections between variables or features, with the ability to enhance biological and microbiological research^9–11^. Utilizing ML methods can support exploratory analyses as part of the feature selection process in determining which variables are important for a given model or question. The method of relative ranked importance is one such concept that can enhance insights into biological relationships in the context of these predictive models^11–13^. While ML approaches have been frequently employed in the context of seasonal IAV in humans^14–16^, there are a paucity of models developed in the context of risk assessment of novel and emerging viruses with pandemic potential, which would necessitate reliance on in vivo model data. Previous research showed that ML models employing both molecular and/or in vivo-derived features for classifying a binary outcome worked well for lethality, but not transmission when validated with additional external data^13^. This could be due to both a lack of understanding of which features are most important to include in the model, and poor labeling of the outcome variable for classification where a better classification or labeling scheme could be more robust and not result in model overfitting.

To improve our knowledge of which virological features are most strongly associated with both RDT and DCT modes, we performed exploratory analyses with an aggregated dataset of 97 IAV spanning both human and zoonotic contemporary IAV. We then used these findings to inform ML algorithms for both transmission modes, including a diversity of molecular determinants, clinical parameters, and infectious titer metrics as input variables. We found transmission mode-specific virological features for both RDT and DCT, showing that intermediate (33–66%) transmission results should be differentially interpreted depending on the mode assessed. In agreement, ML classification models optimized to predict a binary yes/no transmission outcome at >33% RDT and >67% DCT were found to have higher performance metrics relative to traditional 50% cutoffs. Collectively, these findings provide novel insight towards robust interpretation of pandemic risk assessment activities, and contribute to ongoing refinements of the value in vivo-generated laboratory data in both RDT and DCT transmission studies can improve human health.

## Results

### Strong statistical association between IAV receptor binding preference and host origin with transmission outcomes across multiple modes

**Fig. 1:**
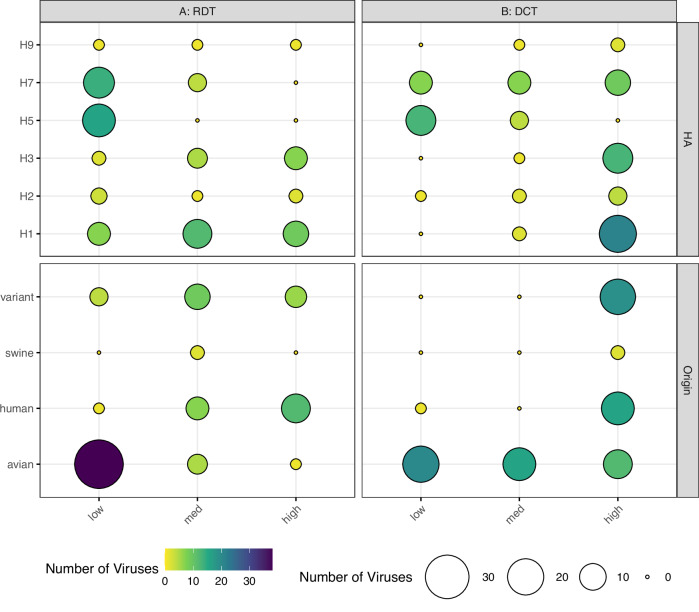
Diversity of influenza A virus subtypes and host origins of strains used in this study. Distribution of IAV in RDT (*n* = 85 unique viruses, **A**) or DCT (*n* = 87 unique viruses, **B**) datasets by viral HA subtype (top panels) or host origin (bottom panels). Host origin was based on virus isolation, not species of isolation (variant: human infection with swine-origin virus). Per-virus transmission frequencies were divided into low (<33%), medium (33–66%) or high (>67%) categories for each transmission mode shown. Both color (purple to yellow) and dot size (large to small) represent number of viruses in the group.

Studies investigating correlates of IAV transmissibility have mainly focused on airborne transmission, neglecting direct and indirect contact transmission modes. To address this gap, we examined the presence of known molecular determinants associated with virus transmissibility in a diverse group of 97 IAV. These viruses were evaluated for their ability to transmit in the presence of direct contact (DCT) or by respiratory droplets (RDT). We classified the transmission frequencies among all ferret donor:contact pairs of each virus as low (<33%), medium (33–66%), or high (>67%) for each transmission mode, among a minimum of three ferret donor:contact pairs assessed per virus, per transmission mode. As expected, transmission frequencies varied by HA subtype, with H5 and H7 viruses exhibiting the lowest frequency in an RDT model, and H1 and H3 viruses exhibiting the highest frequency in a DCT model (Fig. 1A, B). In an RDT setting, avian-origin IAV in the dataset had lower transmissibility than mammalian-origin (inclusive of human, swine, canine, and variant) IAV, which had a more evenly distributed transmission frequency (Fig. 1A). In a DCT setting, avian-origin viruses exhibited an equal distribution of transmission frequencies, whereas mammalian-origin IAV had a relatively high transmission rate (Fig. 1B). Beyond this qualitative assessment, we employed a binary data split (consistent with these types of studies and what is feasible to maintain high sample sizes, we divided viruses associated with >50% or ≤50% transmissibility in both transmission modes) to assess the significance of these associations. Fisher’s Exact tests supported that mammalian-origin viruses, and non-HPAI (highly pathogenic avian influenza) viruses, were strongly associated with >50% virus transmissibility in both transmission modes (Table 1).

**Table 1:** Frequency of >50% virus transmission in either RDT or DCT models

			RDT^a^		DCT^b^	
Feature	Variable 1	Variable 2	*p* value	Odds ratio^c^	*p* value	Odds ratio^c^
Origin	mammalian	avian	3.953e-9	34.56 (7.29, 335.17)	5.926e-9	32.25 (6.92, 306.39)
HPAI	no	yes	1.165e-4^d^	19.41 (2.81, 841.86)^d^	1.152e-7	16.40 (4.81, 67.77)
RBS	H + D	A	6.792e-8^d^	40.78 (5.95, 1758.40)^d^	4.592e-12	46.59 (11.21, 288.25)
RBS	H	A + D	4.717e-10	30.04 (7.93, 148.78)	26.597e-8^d^	40.71 (5.97, 1750.79)^d^
PBS	H	A	1.156e-4	9.68 (2.53, 55.73)	2.486e-8	16.82 (5.24, 63.24)
PB2-627	K	E	0.4109	1.61 (0.47, 5.29)	0.5801	1.55 (0.47, 6.27)
Lethality^e^	low	high	0.2629^d^	4.49 (0.55, 208.50)^d^	0.0491	5.66 (0.93, 61.02)
Weight loss^e^	<10%	>10%	0.5346	0.60 (0.16, 2.38)	1	1.02 (0.24, 3.98)
Temp	≥1 °C	<1 °C	1.00	0.92 (0.25, 3.06)	0.1138	2.47 (0.80, 7.88)

^a^RDT, respiratory droplet transmission model. 28/85 viruses included in analysis with >50% transmission (32.9% of all viruses with transmission results).

^b^DCT, virus transmission in the presence of direct contact. 54/87 viruses included in analysis with >50% transmission (62.1% of all viruses with transmission results). See Supplemental Table 25 for per-variable total observations for each transmission mode tested.

^c^Odds ratio reflects higher odds for Variable 1 in all instances (95% confidence interval in parentheses). High transmission is >50%, low transmission is ≤50%.

^d^An adjustment factor of 1 was added to groups indicated (highly pathogenic avian influenza (HPAI), due to no RDT transmission >50% among HPAI viruses; receptor binding preference (RBS), due to no viruses ≤50% DCT with human (H) binding or no viruses >50% RDT with avian (A) binding (D represents dual binding); Lethality, due to no viruses with high lethality >50% RDT).

^e^Lethality as defined as low (≤50%) on a per-virus basis; Weight loss as defined as > or < 10% mean maximum weight loss on a per-virus basis; Temperature as defined as ≥1 °C or <1 °C mean maximum rise over preinoculation baseline on a per-virus basis.

We next assessed the relative contribution of predicted receptor binding preference (RBS) on the frequency of virus transmission in ferrets. IAV were classified as predicted α2-6 (“human”), α2-3 (“avian”) or “dual” binders based on sequence identity at key amino acid residues in the HA (see Methods). Viruses anticipated to bind to α2-6 linked sialic acids had a higher transmission frequency in RDT or DCT models according to Fisher’s Exact tests (Table 1). Interestingly, predicted α2-3 binding was strongly linked with a lack of virus transmission by the airborne route, while predicted α2-6 binding was more strongly linked with enhanced transmissibility in the presence of direct contact (Supplementary Fig. 1). Taken together, predicted binding to α2-6 sialic acids was sufficient for robust transmission in a DCT setting, but not by the airborne route. Predicted binding to α2-3 linked sialic acids was a strong inhibitor of airborne transmission, but not in the presence of direct contact.

Additional molecular determinants had varied effects on transmission outcomes in both settings. Viruses with a predicted human-like polymerase activity (PBS) were associated with higher per-virus transmission frequencies in both models tested, with avian-like polymerase activity associated with no transmission in an RDT model, and human-like polymerase activity associated with increased transmission in a DCT model (Supplementary Fig. 1, Table 1). Notably, enhanced transmissibility was poorly associated with the residue at PB2 position 627, with comparable odds ratios for >50% transmissibility among viruses bearing an E or K at this position (Table 1), supporting the multifactorial nature of polymerase activity. Logistic regressions for >50% transmissibility, inclusive of variables shown in Table 1 with odds ratios >30, found significance for these parameters for DCT, but not RDT transmission (Supplementary Table 26), indicating mode-specific features.

### Viral titer-based summary measurements were strongly associated with transmission outcomes in DCT, but not RDT models

Several studies support a role for viral shedding from the mammalian respiratory tract in transmission outcomes^17–19^, but have not compared relative differences between transmission modes. To this aim, we examined statistical associations between per-virus transmission outcomes (either RDT or DCT) and a diverse panel of data extrapolated from NW specimens. When data were grouped in these analyses, transmission frequencies for each virus were classified as high (>50% per-virus transmission) or low (≤50% per-virus transmission) for each transmission mode.

**Fig. 2:**
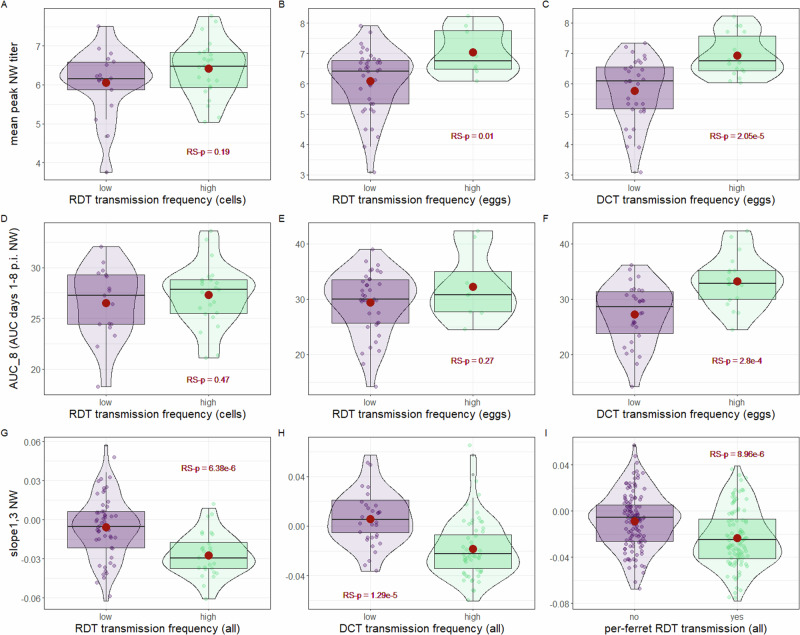
Statistical associations of transmission frequencies and virological measurements in different modes of transmission. Transmission frequencies for each virus were classified as high (>50% per-virus transmission) or low (≤50% per-virus transmission) for each transmission mode (RDT, respiratory droplet titered in cells or eggs, DCT, direct contact titered in eggs). Graphs depict mean peak nasal wash (NW) titer expressed as PFU/ml (cells) or EID_50_/ml (eggs) (**A**–**C**), area under the curve days 1-8 (AUC_8) (**D**–**F**), or the slope between days 1 and 3 (slope_1,3_) (**G**–**I**); slope_1,3_ dataset is inclusive of both egg and cell titration (all). Box and whisker plots specify median plus quartile; red dot depicts mean. Dots represent mean values per virus for all panels except I, which depicts individual ferret transmission outcomes and not virus means. RS-p denotes overall relative statistical significance between groups shown by Welch’s one-way ANOVA (see Supplementary Tables 2, 4, and 6).

We first examined viral titer data from NW specimens collected at defined times post-inoculation (day 1 or day 3 p.i.), or the peak titer recorded (between days 1–5 p.i.). In a RDT setting, NW viral titers in donor animals (following either egg- or cell-titration) did not possess meaningful linear correlations with per-virus transmission frequencies to contact animals (Supplementary Table 1). However, in a DCT setting, linear correlations among egg-titered NW specimens were present against multiple samples tested, with the strongest positive correlation detected with mean peak NW titer [Pearson correlation coefficient (95% CI) *r* = 0.58 (0.37, 0.74), RS-p = 6.31e-6]. In agreement, *t* tests between viruses exhibiting high or low transmission and titers from NW specimens generally did not reach statistical significance in RDT experiments, but did in DCT experiments, notably for mean peak NW titer (RS-p = 2.05e-5, with mean titers of 5.76 and 6.93 log_10_ EID_50_/ml for viruses exhibiting ≤50% or >50% transmissibility in a DCT setting, respectively) (Fig. 2A–C, Supplementary Table 2).

Unlike discrete titers, area under the curve (AUC) measurements permit assessment of virus shedding over defined periods of time. We examined time durations employing ferret NW specimens titered inclusive of the first 4, 6, 8, or 9 days p.i. (AUC_4, AUC_6, AUC_8, and AUC_9, respectively). Independent of the day range employed, robust linear correlations with AUC measurements were present with per-virus DCT, but not RDT, transmission frequency (Supplementary Table 3). Comparable results were observed when conducting *t* tests among viruses stratified by high or low transmission frequency (Fig. 2D–F, Supplementary Table 4), further supporting meaningful statistical associations between AUC measurements of NW viral titer and DCT, but not RDT, outcomes. These results indicate that DCT transmission outcomes are strongly linked with detection of elevated viral titers in NW specimens, but that RDT transmission outcomes are not.

### Infection progression parameter slope_1,3_ strongly associated with transmission outcomes in both DCT and RDT models

Infection progression parameters, such as the growth or decay of NW viral titer between two timepoints, can provide valuable insight regarding the relative fitness and capacity of IAV to replicate over time. In example, we recently showed that NW titers of viruses well-adapted to replicating in the ferret upper respiratory tract (e.g., mammalian-origin IAV) frequently peak day 1 p.i. before dropping day 3 p.i., whereas NW viral titers of avian-origin IAV (which are less likely to bind efficiently to the α2-3 linked sialic acids present on the epithelia of the mammalian upper respiratory tract) are often still increasing between days 1 and 3 p.i.^12,20^. As mammalian-origin IAV are more frequently associated with efficient transmissibility in the ferret model (Fig. 1), we examined if this infection progression parameter was associated with transmission outcomes.

The rate of titer growth or decay in NW specimens between days 1–3, 3–5, or 5–7 p.i. (slope_1,3_, slope_3,5_, and slope_5,7_, respectively) was calculated for each virus assessed in either a DCT or RDT transmission setting. Strong negative correlations were observed against slope_1,3_ and either RDT [*r* = −0.56 (−0.69, −0.39), RS-p = 4.23e-8] or DCT [*r* = −0.47 (−0.62, −0.29), RS-p = 2.42e-6] transmission frequencies, or when viruses were grouped by high or low RDT (RS-p = 6.38e-6) or DCT (RS-p = 1.29e-5) outcomes (Fig. 2G, H). The high statistical significance observed for RDT was maintained when comparing slope_1,3_ and transmission outcome on either a per-ferret or per-virus basis (Fig. 2G, I). In contrast, meaningful linear correlations or statistically significant associations were not present when employing rates of titer growth beyond day 3 p.i. (either slope_3,5_ or slope_5,7_, Supplementary Tables 5, 6). In summary, while NW viral titer measurements (spanning discrete viral titers and AUC-based measurements) were more strongly associated with DCT but not RDT transmission outcomes, the derived quantity slope_1,3_ was meaningfully and statistically linked with both RDT and DCT transmission outcomes. This indicates that viruses that appear to be better adapted for fast, high-titer replication in the mammalian upper respiratory tract (as defined by reaching peak titers earlier p.i.) are more frequently associated with virus transmissibility in both transmission modes.

### Contextualization and interpretation of “intermediate” transmission relative to the mode of transmission evaluated

Ferret studies with IAV typically include a sample size of *n* = 3 or *n* = 4 pairs^21,22^. While transmission outcomes of 0% or 100% transmissibility are straightforward to interpret, there is a paucity of available information pertaining to how to interpret “intermediate” transmission outcomes (e.g., 1/3 or 2/3 transmission events for a given virus). Analyses conducted above were stratified as high or low transmission based on a 50% split, but a close examination of the best inflection point to bifurcate transmission frequency for predictive purposes has not been performed. To explore the most statistically supported way to interpret intermediate transmission events, and to ascertain if this interpretation was dependent on the transmission model employed, we separated per-virus transmission frequencies into low (<33%, or 0/3 or 1/4 transmission events), medium (33-66%, or 1/3, 2/3, or 2/4 transmission events) or high (>67%, or 3/3, 3/4, or 4/4 transmission events) and evaluated data trends for both titer-derived (mean peak NW titer, AUC days 1-8 p.i.) and infection progression parameters (slope_1,3_). Analyses were limited to three groups to increase granularity and improve biological relevance without overdiluting sample size.

**Fig. 3:**
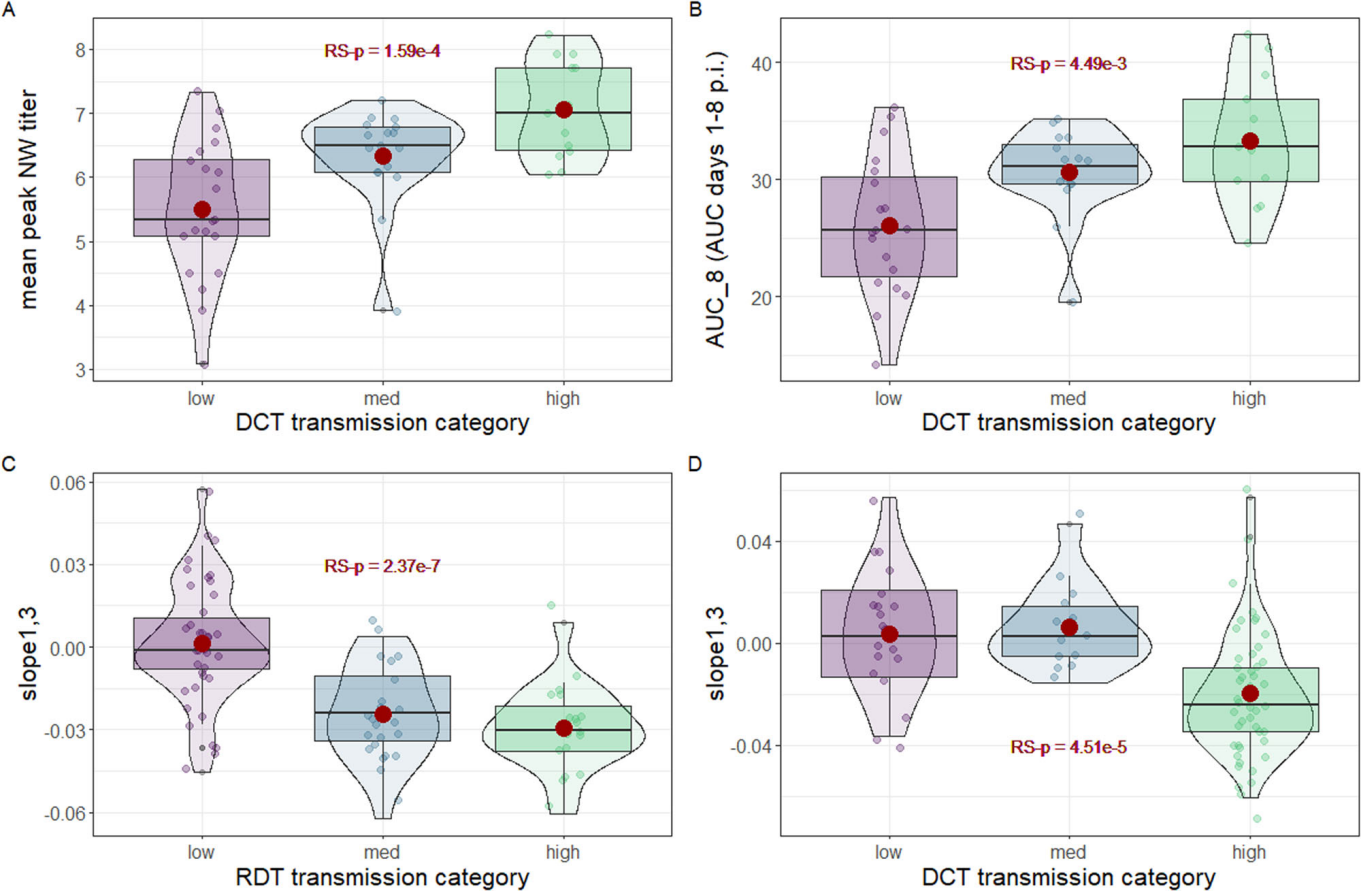
Statistical associations of virological measurements associated with different transmission frequencies and modes. Per-virus transmission frequencies were divided into low (<33%), medium (33-66%) or high (>67%) groups for each transmission mode shown. Graphs depict mean peak nasal wash (NW) titer (**A**) and area under the curve (AUC days 1-8 post-inoculation (p.i.)) (**B**) for direct contact transmission (DCT), and the slope between days 1 and 3 (slope_1,3_) for respiratory droplet (RDT) (**C**) and DCT (**D**) transmission. Box and whisker plots specify median plus quartile; red dot depicts mean. Dots represent mean values per virus for all panels. RS-p denotes overall relative statistical significance between all groups shown by Welch’s one-way ANOVA (see text for additional pairwise comparisons).

We first assessed if employing different categorical classifications of transmission would improve the statistical strength of transmission relationships employing viral titer data. In an RDT setting, bifurcating transmission results at 33% or 67% transmission efficiencies did not meaningfully improve the generally poor statistical associations previously examined at 50% transmission when employing viral titer data (Supplementary Tables 2, 4). In contrast, in a DCT setting, both mean peak NW titer and AUC-based measurements increased with transmission category (Fig. 3A, B), though statistical associations when viruses were bifurcated at 33%, 50%, or 67% transmission efficiency were generally comparable (Supplementary Tables 2, 4).

In contrast to viral titer-based measurements, a strong demarcation was observed when assessing slope_1,3_. In an RDT setting, viruses with low (<33%) transmission frequency had slope_1,3_ values significantly higher than either medium (50%, *p* = 2.2e-5) or high (>67%, *p* = 1.73e-6) outcomes (Fig. 3C). In contrast, in a DCT setting, viruses with high transmission frequencies had slope_1,3_ values significantly lower than either low (*p* = 3.86e-3) or medium (*p* = 4.83e-4) transmission frequencies (Fig. 3D). In agreement, bifurcating RDT transmission frequency at 33% and not 50% increased statistical significance to RS-p = 1.02e-8 (compared to Fig. 2G), and bifurcating DCT transmission frequency at 67% and not 50% increased statistical significance to RS-p = 3.46e-6 (compared to Fig. 2H). Collectively, these results indicate that “intermediate” transmission (e.g., 1/3 or 2/3 transmission outcomes) groups more closely with low transmission events in a DCT setting, but with high transmission events in an RDT setting. In other words, the DCT model offers highest predictive utility when IAV exhibit a high transmissibility (>67%) phenotype, but even ‘inefficient’ transmission results from an RDT model (>33%) can represent an enhanced predictive capacity for IAV airborne transmissibility.

### Machine learning approaches support the highest predictive utility of 33% RDT and 67% DCT transmission outcomes

**Fig. 4:**
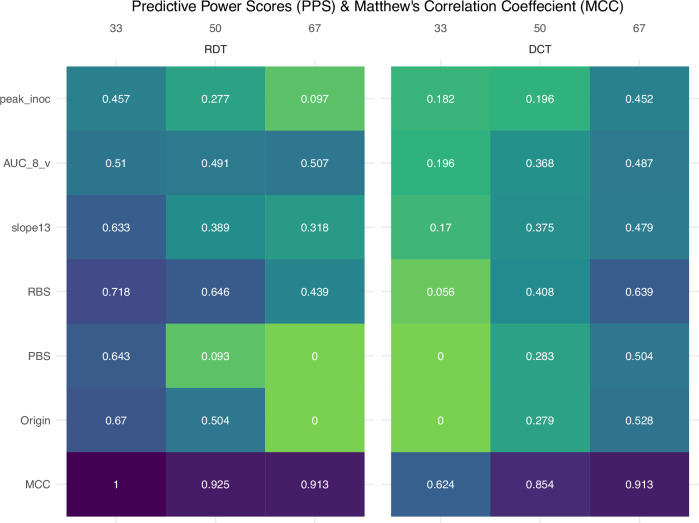
Comparison of predictive power scores (PPS) and Matthew’s Correlation Coefficient (MCC) when employing different gating thresholds for RDT and DCT models. Predictive power scores were determined for a panel of titer-based (peak inoculated titer (peak_inoc), area under the curve days 1–8 per virus (AUC_8_v), slope between days 1 and 3 (slope_1,3_)) and non-titer based (receptor binding preference (RBS), polymerase activity (PBS), virus Origin) measurements were assessed for each gating threshold (33, 50, and 67%). Values range from 0 (worst, green) to 1 (best, purple). Matthew’s Correlation Coefficient was determined for ML models employing each gating threshold. Values range from −1 (no agreement) to 1 (agreement).

To further assess the predictive implications of bifurcating “intermediate” transmission events using different gatings, we examined predictive power scores (PPS) for each gating threshold (33, 50, and 67%) for a panel of titer- and non-titer-based measurements in the context of both RDT (Supplementary Table 7) and DCT (Supplementary Table 8). PPS is a nonlinear alternative to correlation using a normalized index (0 to 1) to show how much a variable could be used to predict an outcome. For every numerical parameter associated with viral load (peak_inoc, AUC_8_v, slope_1,3_) or categorical viral property (RBS, PBS, Origin) tested, the highest predictive power was present when transmission outcomes were bifurcated at 33% for RDT, and 67% for DCT (Fig. 4), in agreement with statistical analyses shown above. Furthermore, we found that bifurcating transmission results at 33% in an RDT setting (and not 50% or 67%), and 67% in a DCT setting (and not 33% or 50%) resulted in logistic regression models that were the most predictive (with the lowest AIC weights [172.73, 150.44]) and explanatory (with the highest Tjur’s *R*^2^ values [0.67, 0.70]) when employing variables with the highest odds ratios shown in Table 1 (Supplementary Table 26). Beyond logistic regression, use of elastic net regularization regression analysis supported many of the findings (Supplementary Table 9). Elastic net multivariate regression is a statistical technique using penalties to model coefficients for a balanced method of variable selection and shrinking multicollinearity among several feature variables, which can control for the influence of confounding factors when included as predictors in the model. Common features were also identified using the reverse feature selection method, which iteratively removes features based on predictive importance. This approach further supported the consistent association of Origin and slope_1,3_ with RDT outcomes, and MBAA, Origin, HA/Subtype, and slope_1,3_ with DCT outcomes.

Overall, these results lead us to test these features in random forest machine learning models (Supplementary Table 10), to further investigate predictive importance using a different multivariate approach. We consistently included Origin, RBS, and PA in all RDT and DCT models. Defining subtype using HA by itself (not in combination with NA) was found to be more predictive. Furthermore, we explored the inclusion of MBAA, AUC_8, peak_inoc, and slope_1,3_ (on a per-virus or per-ferret basis, and as a per-virus category), finding slope_1,3_ per virus as both a numeric titer and a categorical feature worth pursuing. Hence, final model features were Origin, RBS, PBS, HA subtype, and slope_1,3_ per virus (either numeric or categorical) for RDT and DCT, a total of four models (Supplementary Table 10).

**Fig. 5:**
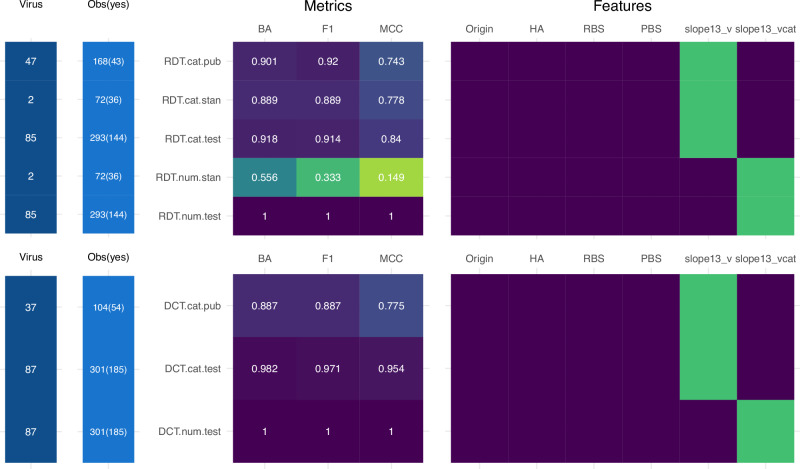
Comparison of transmission model performance metrics and feature selection iterations. The number of unique total viruses in the testing and training datasets (Virus), and binary observations based on the classification model tested (Obs(yes)), for each model iteration shown. Heat map depicting balanced accuracy (BA), F1 score (F1), and Matthew’s Correlation Coefficient (MCC) performance metrics for respiratory droplet transmission (RDT, top) or transmission in the presence of direct contact (DCT, bottom) models employing different feature selections. Models employ either a categorical (.cat) or numerical (.num) slope_1,3_ variable (slope between days 1 and 3), and employ either in-house data (.test), standardization exercise data (.stan), or published literature (.pub). Values range from 0 (worst, green) to 1 (best, purple) for BA and F1, and from −1 (no agreement) to 1 (agreement) for MCC. Feature inclusion for all RDT (top) or DCT (bottom) models shown. Purple, feature inclusion; green, feature exclusion. Individual feature definitions tested are provided in Supplementary Table 10. Full scope of all model metrics for models are reported in Supplementary Table 21.

Previous efforts from our group to develop an ML model to predict RDT transmission employed a 50% transmission event bifurcation of results, which turned out to be not very predictive^13^. We also did not test models for DCT. Based on exploratory analyses of each gating threshold, we hypothesized that predictive models employing a 33% bifurcation for RDT and 67% for DCT would be more robust than using 50% for both models. To test this, we ran models with all three transmission event gating thresholds (33, 50, 67) and compared performance using Matthew’s Correlation Coefficients (MCC) as a metric of success. Overall, we found that using the transmission gating threshold of 33% for RDT models, and 67% for DCT resulted in consistently higher metrics. This tracks with the pattern we found using predictive power scores (Fig. 5) and supported by MCC being the highest for RDT_33 (0.84) and DCT_67 (0.954). Full model performance metrics for all training data models and algorithms tested are in supplementary tables for RDT (Supplementary Table 11) and DCT (Supplementary Table 12). Full model performance metrics for all untuned testing data models and algorithms are also in supplementary tables for each RDT (Supplementary Tables 13–15) and DCT (Supplementary Tables 16–18) transmission gating thresholds (33, 50, 67). We also present the confusion matrices (the number of true/false positive/negative predictions) and MCC for all untuned testing data models and algorithms for RDT (Supplementary Table 19) and DCT (Supplementary Table 20).

### High performance metrics of ML models employing RDT_33 and DCT_67 against internal and external datasets

Once establishing that RDT_33 and DCT_67 were the most robust, we performed fine tuning of both models to optimize performance. To improve applicability to the field, we assessed slope_1,3_ as both a numerical and categorical value (positive, zero, negative). We assessed performance of the final tuned random forest model for RDT_33 against three independent datasets: CDC internal test data withheld from initial model training, data from a worldwide ferret IAV transmission standardization exercise, and data aggregated from the published literature (see “Methods”). Models including either numerical or categorical classifications of the slope_1,3_ feature were found to be of high performance, though this differed with the dataset tested. Employing CDC data, high performance metrics (notably balanced accuracy, F1, and MCC) were detected with either numerical (RDT.num.test) or categorical (RDT.cat.test) classifications (Fig. 5, Supplementary Table 21). However, use of standardization data to validate models revealed high-performance metrics for the categorical (RDT.cat.stan) but not numerical (RDT.num.stan) classification, where we find a high number of false negatives (more “yes” predictions). This is consistent with initial findings employing the numeric feature AUC in prior RDT transmission model development^13^, where derived quantities of titer values appear to be very predictive but fail to hold up with additional data testing; this is likely due to noise in the data causing overfitting. Stringent validation of this model with independent data aggregated from the published literature (RDT.cat.pub) yielded comparably high-performance metrics as the other two datasets. These results support that a model predicting >33% IAV transmissibility in an RDT setting can yield high performance that are maintained when evaluated with independently collected data, employing an infection progression parameter that can be extrapolated from relative changes in viral titer over time.

When examining the final tuned random forest model performance for DCT_67, we find similar trends to RDT (Supplementary Table 21). Models including either a numerical (DCT.num.test) or categorical (DCT.cat.test) slope_1,3_ variable had high-performance metrics (Fig. 5). Stringent validation of this model employing a categorical slope_1,3_ variable with independent data aggregated from the literature (RDT.cat.pub) also yielded high-performance metrics. Sufficient independent data for validation of a DCT model employing non-CDC data with a numerical slope_1,3_ variable was not available, though it is likely that (similar to the RDT model shown here and previously^13^) diminished performance would also be observed if conducted. These results support that a model predicting >67% IAV transmissibility in a DCT setting can yield high performance metrics that are maintained when evaluated with independently collected data. Furthermore, these models support that contextualization of intermediate transmission events relative to the mode of transmission evaluated can yield high predictive utility.

### Commonalities in highest-ranked importance features across RDT and DCT models

**Fig. 6:**
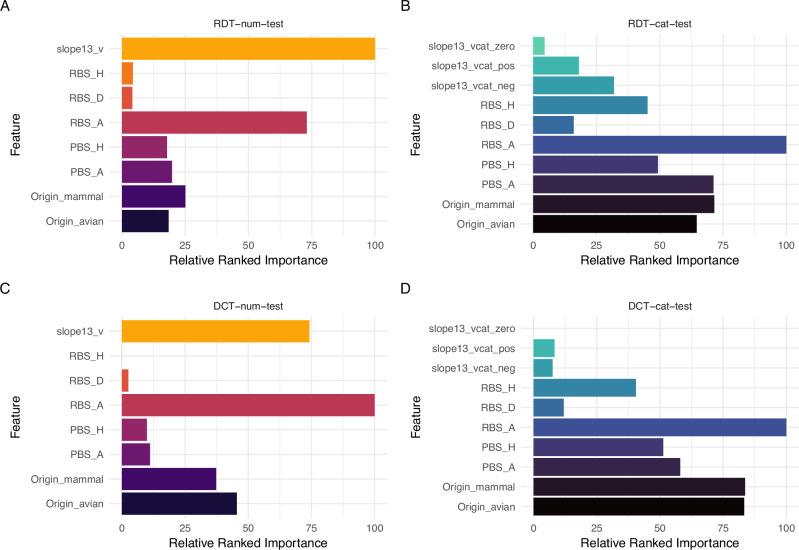
Variability in feature selection among RDT and DCT models employing different features. Relative ranked importance of top numeric features included in respiratory droplet (RDT) and direct contact (DCT) transmission models employing a numerical (v; **A**, **C**) or categorical (vcat; **B**, **D**) slope_1,3_ variable (slope between NW days 1 and 3 p.i.). Categorical slope_1,3_ is designated as either being positive (pos), negative (neg) or neutral (zero). Virus Origin is designated as being either avian or mammal. Receptor binding preference (RBS) is designated as being avian (A), human (H), or dual (D); predicted polymerase activity (PBS) is designated as being avian (A) or human (H). Relative ranked performance among features of all models, including HA subtype which is not presented in these graphs, is shown in Supplementary Table 22. Relative ranked importance values are set to 100 for the most important feature and scaled to relative importance for remaining features independently within each model. For each model features are consistently scaled but separated out for visual purposes, and not meant to convey direct feature comparisons, only relative ranking.

While both RDT and DCT transmission models contained the same panel of features (Fig. 5), we explored if relative feature importance for each model varied. The features found most important for model predictions were relatively consistent between RDT_33 and DCT_67 when containing either a numeric or categorical slope_1,3_ (Fig. 6, Supplementary Table 22). With a numeric slope_1,3_, it was the most important feature for RDT_33 (Fig. 6A), and the second most for DCT_67 (Fig. 6C). In both models, a predicted avian RBS was the other highest ranked feature detected (ranked second and first for RDT and DCT models, respectively). Ranked relatively lower for both models were Origin and PBS, followed by HA subtype. In contrast, both RDT and DCT models including a categorical slope_1,3_ had this feature ranked comparatively lower (Fig. 6B and D, respectively), whereas an avian RBS was maintained as the top ranked feature, followed by Origin and PA. In the DCT_67 model, HA subtype takes on a bit more importance than slope_1,3_ compared to the RDT_33 model. Complete relative ranked importance values generated on training data for all models and algorithms are available for RDT (Supplementary Table 23) and DCT (Supplementary Table Variable 24). Collectively, these findings support the crucial role a predicted avian receptor binding preference (RBS) contributes to the high-performance metrics observed in all transmission models tested.

## Discussion

Improving the rigor of small mammalian models employed for pandemic risk assessment can augment the utility of the conclusions drawn from these experiments. As use of the ferret model to evaluate the pandemic potential of IAV has escalated over the past two decades, periodic assessment of how these data are interpreted and contextualized is warranted, to ensure responsible and rigorous interpretation of contributing laboratory studies to these risk assessment rubrics^23,24^. By aggregating transmission results from >450 unique ferret transmission events, we were able to critically assess which features (based both on viral sequence-based information, and following in vivo replication) were most strongly statistically linked with transmission outcomes in two different settings, while overcoming some of the challenges of small sample size variability in any one experiment^21,22^. These studies identified that transmission events in the presence of direct contact and following exposure to respiratory droplets differ in which data collected from donor animals are most predictive of a successful transmission outcome. Furthermore, these analyses supported that “inefficient” or “intermediate” transmission events should be contextualized relative to the transmission mode assessed. Employing machine learning (ML) approaches using both internal data and external, independently generated datasets, collectively support the biological relevance of the conclusions drawn, and provides support that ML algorithms can offer supportive evidence towards refinements of existing laboratory protocols and procedures. Collectively, this work contributes to overcoming limitations present when conducting independent ferret transmission experiments with small sample sizes^21^, by responsibly interpreting results across multiple studies to identify virological and within-host properties meaningfully associated with frequency of transmission outcomes not readily identifiable when conducting small studies in isolation which can subsequently inform and improve these discrete experiments^22^.

Features assessed in this study were restricted to key parameters known or presumed to modulate pathogenicity and transmissibility in mammals^2,12,25^. Influenza A viruses frequently replicate throughout the upper and lower mammalian respiratory tract, including the lung; our analyses focused on serially collected nasal wash (NW) specimens and not tissues collected at a fixed time during scheduled necropsy. Evaluation of nasal turbinate, trachea, and lung tissue day 3 p.i. found a lack of statistical correlation with either clinical or transmission outcomes (Supplementary Table 1). NW specimens are inclusive of virus replication at multiple anatomical locations^12^ and as such may be more representative than a single tissue for this purpose. It is also possible that day 3 p.i. represents an appropriate timepoint for comparative assessments of viral pathogenicity, but not transmissibility; a closer examination of other time intervals during the acute phase of infection (especially collected on days 1–2 p.i.) may reveal enhanced utility of tissue data for these purposes that were not apparent in our analyses^18,26^. While molecular determinants assessed were limited to predicted receptor binding preference and predicted polymerase activity, inclusion of full protein sequence (from the HA alone, or all gene segments) could provide further predictive strength of ML algorithms and identify previously unrecognized determinants of viral transmissibility, as could incorporation of other determinants such as HA stability as features in the models themselves^27^. Clinical parameters (lethality, weight loss, temperature rise) were not strikingly associated with either RDT or DCT transmission outcomes (Table 1), and were not among the highest-ranked features ultimately included in ML models (Fig. 5), though further investigation of the relative contribution disease severity in the context of virus replication contributes to transmission outcomes would be of interest.

Viral burden and time of exposure have both been identified as critical parameters in mediating transmission events in vivo. Higher viral loads in the mammalian upper respiratory tract have been linked with increased airborne transmission in ferrets and guinea pigs^17–19^, though these studies have varied in their measures of viral burden (infectious virus or viral RNA at a fixed time point, or area under the curve measurements), and have not assessed relative differences across multiple transmission modes. In airborne transmission settings, early timepoints (within the first 1–3 days) post-inoculation represent a critical interval for transmission to occur, as supported by the relatively brief duration of exposure needed to support transmission^28–30^ coinciding with the days of highest release of viral-laden aerosols shed by inoculated animals^19^. Our results are generally consistent with these findings, and with use of the infection progression parameter slope_1,3_, provide greater insight into how viral replication and time work in tandem to collectively shape transmission outcomes. Viruses with a negative slope_1,3_ are more likely to be of mammalian-origin, more likely to have the ability to bind α2-6 linked sialic acids, more likely to achieve peak titers more rapidly in the ferret upper respiratory tract, and more likely to transmit onward (in either a direct contact (DCT) or respiratory droplet (RDT) transmission setting), than viruses with a positive slope_1,3_. Continued investigation of how timing of peak detection of virus replication in the upper respiratory tract contributes to subsequent transmission outcomes is warranted.

Our finding of improved predictive outcomes employing at 33% RDT and 67% DCT models supports that “intermediate” transmission events detected during RDT and DCT experiments occur for different reasons. For DCT, “inefficient” transmission events may be driven by high viral titers in inoculated animals, resulting in sporadic infection of contact animals due to the close, sustained contact of these animals in this model system and diversity of potential transmission modes encapsulated by the DCT model^7^. Pair-housing of animals represents a form of enrichment that may result in greater activity levels than animals housed individually^31^, and it is possible that DCT transmission outcomes <67% are driven primarily by stochastic interactions between animals (possibly following exposure to contaminated fomites), and do not necessarily reflect the true transmission potential of a virus in this setting, but are rather a reflection of the high variability that can be present between inoculated animals in shedding virus into the environment. In contrast, the stringent nature of the RDT transmission model may result in fewer inadvertent transmission events, supporting that even a 33% transmission outcome represents a meaningful event. This differential bifurcation may further support a limitation of the DCT model (in that transmission events <67% are not reliable, and only >67% transmission outcomes are meaningful in the context of risk assessment), while concurrently showing that intermediate transmission events are more predictive of transmission potential when assessed in a RDT setting.

Validation of ML models with external data is an important step to assess model performance and generalizability. External data not fully consistent with the internal training data (especially that generated by multiple independent groups) provides a real-world scenario and can help identify potential limitations or biases in the model^32^. In our case, external validation consistently supported the model, showcasing informative utility of the findings more broadly, despite the unavoidable limitations of training models on data from a single laboratory which may not capture the full scope of heterogeneity present across laboratories worldwide performing risk assessment work of this nature. The findings of machine learning highlight the usefulness of slope_1,3_ in transmission event detection, and the splitting of predictive transmission events to >33% RDT and <67% DCT. Variable importance from ML models is also consistent with exploratory data finding predicted receptor binding preference consistent in both transmission models, and viral host origin slightly more important for DCT. The predictive ML findings generally support the associative exploratory findings, helping to reinforce the overall conclusions of this study. ML results also illustrate the issue of model overfitting in the case of a numerical slope_1,3_, or AUC as previously found^13^. By transforming this data to categorical we were able to overcome issue of overfitting, even if losing some specificity of the data. However, smoothing out this noise is warranted in this case, and further exploration of categorical groupings or thresholds is justifiable.

This work was designed to encompass the most frequently employed experimental protocols for in vivo assessments of virus transmissibility employing the ferret model^33^, but nonetheless includes limitations inherent in the field. Models are specific to experimental inoculation of serologically-naive ferrets; while data from these experiments can align with IAV secondary attack rates in humans^8^, results cannot be extrapolated as such. All three internal and external datasets employed in this study included multiple titration matrices for viral titer determination; it should be noted that the slope_1,3_ feature is independent of viral units^20^. Models were trained on data generated from NW specimens, and the external published dataset included categorical slope_1,3_ values derived from NW collection only; while the standardized dataset included both serially collected NW and swab specimens^33^, extrapolation of results from this work to studies collecting NW specimens under different conditions and/or using swabs to assess replication in the upper respiratory tract should be undertaken with caution. While it is standard practice to collect NW on alternate days post-contact, we were only able to include studies in the published dataset employing a categorical slope_1,3_ that collected specimens on days 1 and 3 p.i., and not days 2 and 4 p.i.; subsequent investigation of the potential utility of a slope_2,4_ infection progression parameter could be informative. Results are specific to 1:1 donor:contact ratio experiments following continuous exposure between donor and contact animals; it is possible that use of abbreviated exposure windows could yield different experimental outcomes and input variables with different feature importance. Predicted receptor binding preference and predicted polymerase activity did not include laboratory assessments confirming these phenotypes, which could improve the utility of these parameters^12^. Virus transmission was defined in this study as a detectable infectious virus titer on at least 1 day post-contact plus seroconversion to homologous virus; we did not assess seroconversion in the absence of infectious detection, and did not explore differences in the timing and/or relative strength of transmission events when detected.

The diversity of modes by which IAV can transmit necessitates a diversity of mammalian models in which to assess the pandemic potential of these viruses as they continue to emerge from zoonotic reservoirs. Both DCT and RDT models assessed here provide valuable information and context to the field, as supported by the plethora of studies employing both transmission modes aggregated for external validation of our findings. First, as these models only utilize data from inoculated ferrets to predict transmission outcomes, initial screening of results generated from experimentally inoculated ferrets may lead to fewer overall animals used to assess transmission phenotypes. Second, by identifying the most highly ranked features included in the best-performing model iterations, this work improves our understanding of the key virologic features associated with successful transmission outcomes in each model, and highlights that results from each model should be carefully considered. Third, by comparing models using different outcome cutoffs (e.g., 33% vs 67% transmission), we can extend our biological understanding of transmission mode-specific interpretation of results so these findings can be appropriately contextualized in risk assessment settings. While DCT transmission events appear to be driven by overall viral load in inoculated animals, with ‘intermediate’ transmission events not very predictive of a high transmission capacity in this setting, RDT transmission events appear to be governed, at least in part, by how quickly a virus can achieve peak titers in the upper respiratory tract, with ‘intermediate’ transmission events offering higher predictive value of high transmission capacity by this route. Continued refinement of existing in vivo models employed for risk assessment will improve the ability of these necessary experimental readouts to support public health efforts.

## Methods

### Dataset sources and description

Three independent datasets were employed in this study. All exploratory analyses and initial ML feature selection work was conducted with a dataset of serially collected observations from IAV-inoculated ferrets generated by the CDC (“internal”) following inoculation with a high (10^5^–10^7^ infectious units) dose of virus (87.7% of ferrets received 10^6^). These data are available on data.cdc.gov and described in further detail elsewhere^12,13,34^. Experiments in the internal dataset were conducted at ABSL2 or ABSL3 containment, including enhancements as required by the US Department of Agriculture and the Federal Select Agent Program^35^, under guidance of the CDC IACUC. Virus transmissibility was assessed in the presence of direct contact (DCT, with inoculated and contact animals co-housed) or via respiratory droplets (RDT, with inoculated and contact animals in adjacent cages with perforated side walls to prohibit direct and/or indirect contact) at a strict 1:1 donor contact ratio as described previously^36^; in both transmission settings, transmission was defined by the presence of virus in contact animal NW specimens and seroconversion to homologous virus at the end of the experiment (approx. day 21 post-contact).

Two external datasets were employed for validation of ML algorithms. One external dataset consists of data from a standardization exercise (“standardized”)^33^, which evaluated RDT transmission of two H1 subtype IAV; analyses shown in this study employed the non-normalized viral titers reported directly from contributing groups. The second external dataset consists of data previously published in the literature (“published”, excluding all experiments present in internal dataset) for both RDT or DCT transmission assessments, that met our inclusion criteria for compilation^13^. Inclusion criteria were: source publications were PubMed-indexed, ferrets were healthy and serologically naïve to circulating IAV prior to inoculation, inoculation was performed with a high (10^5^–10^7^ infectious units) dose of IAV delivered intranasally in a 0.5-1 ml volume, ferrets remained in contact for the duration of the experiment, transmission experiments reporting >0% transmission were conducted at a 1:1 donor:contact ratio, and sequence data matching the virus strain name was publicly available (NCBI or GISAID). If a group reported 0% transmission in a DCT setting, then 0% transmissibility by RDT was extrapolated, and if a group reported 100% transmission in an RDT setting, then 100% transmission by DCT was extrapolated.

### Analysis overview

All analyses were conducting in R v4.2.1 using the packages tidyverse v1.3.2^37^ and funModeling v1.9.4^38^. Figure creation used ggplot2 v3.4.0^39^, GGally v2.1.2^40^, ggpubr v.0.5.0^41^, ggstatplot v0.10.0^42^, and patchwork v1.2.0^43^. All viral titers are presented as log_10_ titer, and all calculations were performed with the log_10_ of the measured virus titer. For viral titer data in the internal dataset, titration of specimens for the presence of infectious virus was performed in either embryonated hens’ eggs (expressed as 50% egg infectious dose (EID_50_)) or standard plaque assay in MDCK cells (plaque forming units (PFU)). Area under the curve (AUC) was calculated as previously described^12^. Statistical analyses used ggstatsplot version 0.10.0, correlation tests and Fisher’s exact tests using base R stats. Machine learning analyses used the caret v6.0-94 package^44^, with fastDummies v1.6.3^45^ for dummy variable creation, and rsample v1.2.0^46^ for splitting the data into a 70% training and 30% testing dataset. Additional R packages used as listed below in the corresponding methods sections.

### Statistical analyses

Pearson product-moment correlations (continuous vs continuous variables) or Biserial correlations (for continuous vs dichotomous variables) were calculated without adjustment for multiple comparisons, and p values are reported as a ranking statistic (RS-p), which provides a relative measure of the statistical strength used to compare different associations. Pearson correlations were considered moderate (>0.4) or weak (<0.4). Welch’s *t* tests and one-way ANOVA were calculated when comparing continuous variables (such as viral titer) to categorical measures. Odds ratios for Fisher’s exact tests were considered strong (>15), moderate (>3), or weak (<3).

### Machine Learning inputs and outputs—feature selection, RDT/DCT thresholds

R code is available on Github (https://github.com/Troy-Kieran/machine-learning-influenza-transmission-ferret)); building off a previously established respiratory droplet transmission (RDT) model^13^ we implemented three difference gating strategies for both the RDT and direct contact transmission (DCT) model. For each respective model we used the percent transmission on a per virus basis to determine a transmissible event (yes) or not (no). We employed a threshold at greater than 33, 50, or 67% transmission to be “yes”. To extend observations where experimental data for RDT or DCT was missing, we set DCT transmission at 100% if RDT was 100%, and we set RDT at 0% if DCT equaled 0%.

Using the previous model^13^ as a starting point, we employed a few different methods for feature selection for each gating strategy for prediction of the binary classification outcome of transmission yes or no. We used the ppsr v0.0.2 package^47^ to examine predictive power scores, the glmnet v4.1-6 package^48,49^ to perform elastic net regularization on a binomial logistic regression using an alpha of 0.35 and 10 cross fold validation to determine the best lambda. The best lambda model was run, and coefficients checked using the coef function in stats v4.2.1 package to examine the degree of feature importance. We also used the predict function in stats evaluating with the root mean square error (RMSE) from the Metrics v0.1.4 package^50^ to assess overall feature sets for machine learning feature inclusion. Additionally, we employed backward feature selection using the rfe function from the caret package. Collectively these methods gave us a sense of the overall feature space of which variables we tested in machine learning models.

### Model testing and evaluation

Following our previous study^13^ we tested 5 machine learning algorithms that consistently had better performance; neural network (nnet), gradient boosting machine (gbm), random forest (ranger, rf), and support vector machine (svm). We evaluated models and algorithms using the 70–30% internal training-testing dataset split with two repeats of 10 cross fold validation in the caret trainControl function. We calculated 14 model metrics and assessed performance using balanced accuracy, F1 score, and Matthew’s Correlation Coefficient (MCC). Relative ranked importance of features in each model was calculated using the varImp function from caret, scaling the importance value so 100 was set for the most important feature.

The random forest algorithm was consistently well performing, and we used this for our final RDT and DCT feature set models. Once a final set of features for a model was determined we hyperparameter tuned the mtry parameter of random forest in the caret train function using balanced accuracy for best model prediction. Final model performance was assessed with balanced accuracy, F1 score, and MCC. We validated our tuned models using the internal testing data, and two external datasets (standardization and published).

## Supplementary information

Supplemental Material

Supplemental_Tables

## Data Availability

Data is available from data.cdc.gov under the title “An aggregated dataset of serially collected influenza A virus morbidity and titer measurements from virus-infected ferrets”. A companion data descriptor providing more data is published^34^.
