# Supplementary material for: Optimal thresholds and key parameters for predicting influenza A virus transmission events in ferrets
Source: Npj Viruses. 2024 Dec 9;2:64. doi: 10.1038/s44298-024-00074-w (PMC11628394; doi:10.1038/s44298-024-00074-w)
Supplement: Supplementary file 1 — Supplemental Material [file 44298_2024_74_MOESM1_ESM.pdf]

Supplemental Material

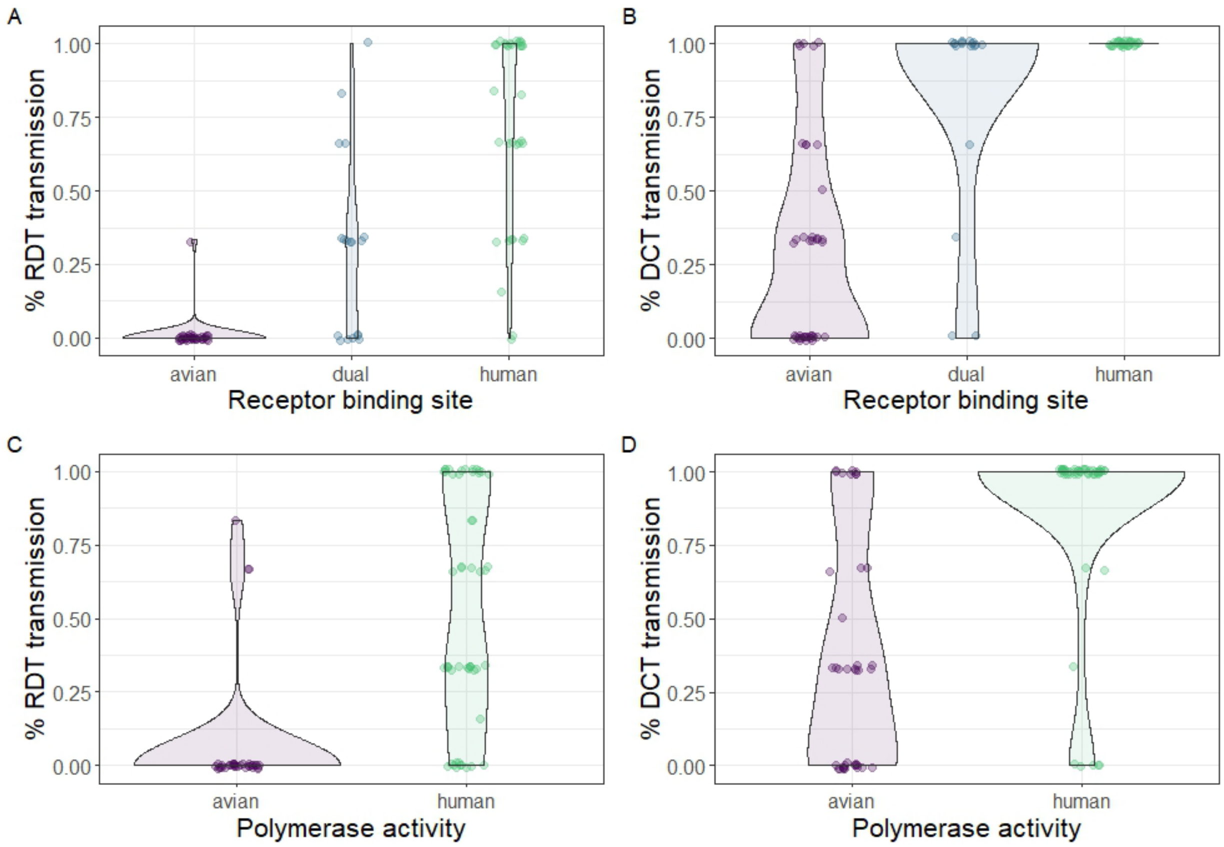

Supplemental Figure 1. Molecular determinants associated with transmission frequencies in different modes of transmission. Transmission frequencies for each virus were classified as high (>50% per-virus transmission) or low (≤50% per-virus transmission) for each transmission mode (via respiratory droplets (RDT) or in the presence of direct contact (DCT)). Graphs depict per-virus RDT or DCT transmission frequency among viruses displaying either avian-like, human-like, or dual predicted receptor binding site preference based on specific residues in their HA surface receptor (A, B) or avian-like or human-like polymerase activity based on specific residues in PB2 (C, D).
